# A Smartphone Application Designed to Engage the Elderly in Home-Based Rehabilitation

**DOI:** 10.3389/fdgth.2020.00015

**Published:** 2020-09-08

**Authors:** Thelma Androutsou, Ioannis Kouris, Athanasios Anastasiou, Sotiris Pavlopoulos, Fariba Mostajeran, Doris-Eva Bamiou, Gregory J. Genna, Sergi G. Costafreda, Dimitrios Koutsouris

**Affiliations:** ^1^Biomedical Engineering Laboratory, National Technical University of Athens, School of Electrical and Computer Engineering, Athens, Greece; ^2^Department of Informatics, Human-Computer Interaction, University of Hamburg, Hamburg, Germany; ^3^University College London, UCL Ear Institute and UCLH Biomedical Research Centre, National Institute for Health Research, London, United Kingdom; ^4^Division of Psychiatry, University College London, London, United Kingdom

**Keywords:** mHealth, smartphone, motivation, elderly, rehabilitation, user experience

## Abstract

As life expectancy increases, it is imperative that the elderly take advantage of the benefits of technology to remain active and independent. Mobile health applications are widely used nowadays as they promote a healthy lifestyle and self-management of diseases, opening new horizons in the interactive health service delivery. However, adapting these applications to the needs and requirements of the elderly is still a challenge. This article presents a smartphone application that is part of a multifactorial intervention to support older people with balance disorders. The application aims to enable users to self-evaluate their activity and progress, to communicate with each other and, through strategically selected motivational features, to engage with the system with undiminished interest for a long period of time. Mock-up interfaces were evaluated in semi-structured focus groups and interviews that were performed across three European countries. Further evaluation in the form of four pilot studies with 160 participants will be performed and qualitative and quantitative measures will be used to process the feedback about the use of the application.

## Introduction

Advances in medicine and pharmacology, improvement in the quality of life and decline in mortality rates have led to people living longer and being more active than in the past. The population of people over 60 years old is rapidly growing in developed countries and thus a number of social and economic changes need to be addressed in the direction of inclusive societies. In order to maintain social cohesion and to balance the burden on health and care systems, this trend of aging population highlights the need for simultaneous extension of active aging. Elderly people should be enabled to be economically and socially independent and their needs and preferences should be taken into account in the process of designing and offering products and services.

Toward this direction, Information and Communications Technology (ICT) can play a major role in increasing the quality of life of the elders by solving the gap between their wishes and their needs. “Gerontechnology” is defined as the study of technology for ensuring good health, full social participation, and independent living throughout the entire life span, as long as it may extend (1, 2). It includes applications aimed at improving the quality of life, social interactions, medical and psychological support, safety, and cognitive education of the elderly. Among its most dominant sectors is that of mobile communication, due to the rapid development of the technologies and capabilities of mobile devices, as well as the convenience the latter offer to people's daily lives. Mobile health (mHealth) applications, whether referring to stand-alone solutions or parts of an intervention, offer new capabilities and opportunities within health service delivery to patients and clinicians. mHealth solutions oriented to healthy lifestyle and self-management of diseases has attracted much of the research interest in recent years (3).

Despite the fact that, compared to younger people, older adults are much less familiar with technology, research has shown that they are nowadays much more digitally aware than they were in the past. They prefer to use a smartphone than other mobile devices and technological tools and the main functions they are interested in are the capability to communicate and get informed (4). They are also eager to learn how to use smartphones, especially when it comes to caring for their health (5). However, seniors' access to smartphone applications and assistive technology remains a challenge. Unfamiliarity and lack of appropriate training are undoubtedly some of the main obstacles. Nevertheless, an equally important factor in the low rate of technology adoption by the elderly is the exclusion of their needs and requirements when designing and implementing technological solutions (6).

Physical activity plays an important role in maintaining good physical and mental health and its promotion is especially important in the elderly, as it is the most sedentary population subgroup (7). There is a large number of mHealth applications aimed to stimulate physical activity, but most of them are not focused on older people. Advances in technology and capabilities, as well as greater accessibility to the benefits it offers, have led to studies directing to the needs of this target group. In (8) a training app that runs on a tablet and assists, monitors and motivates older people to follow personalized training plans autonomously at home is presented. The application includes both individual and social motivation techniques as well as a virtual training plan community. STARFISH is another smartphone-based application that enables the older users to accurately self-monitor their physical activity (9). Goal setting, action planning, feedback and social support are the main features that have been used in its context and showed potential to increase user acceptance. A smartphone and a heart rate belt were used as a mHealth system in order to monitor and promote elderly peoples' daily activity in a care home setting (10). The participants of this 10-week intervention increased their physical fitness levels. Gamification techniques in mobile applications can also play a quite beneficial role in promoting health in older adult populations. A game that embeds exercise and health education into a game context familiar to older adults was developed and the results of its utilization showed that it can engender high levels of adherence (11). In (12), a personalized behavioral intervention program based on theoretical constructs from the self-efficacy theory was developed. The program included personalized physical activity training, real-time physical activity self-monitoring, interactive prompts, and feedback with a smartwatch, phone consultation with an exercise trainer and research team members, and weekly financial incentives for achieving weekly physical activity goals. The SmartWalk project, on the other hand, focuses on creating a physical activity monitoring system for smart cities, based on the use of mHealth, where older people are motivated toward a physical active lifestyle and health care professionals can supervise them for best results.

This article introduces a smartphone application to support older people with balance disorders. The application was developed as part of the project HOLOBALANCE (HOLOgrams for personalized virtual coaching and motivation in an aging population with BALANCE disorders)^1^, which aims to deliver a radically new cost-effective virtual coach to improve balance, cognition and physical activity. The main goal of the smartphone application is to promote physical activity and the user's engagement in the use of the HOLOBALANCE system. Design and motivation techniques have been incorporated into the application to help the user comply with their treatment plan, keeping the interest undiminished. The rest of the paper is structured as follows. The following section describes the HOLOBALANCE project and its modules. In the third section, the purpose, design and motivation features and evaluation of the Activation Planning Application are described in detail. Finally, conclusions and future steps are presented in the fourth section.

## The Holobalance Project

Human balance is the result of many body systems working together: the visual and vestibular systems, proprioception and the musculoskeletal function are integrated in a complex and multifactorial way. The interaction between these systems and sophisticated mechanisms support anticipatory postural adjustments and adapt to changing environmental and balance task demands by means of sensory re-weighting (13). Age-related decline in all these sensory inputs and functions in older people is well-documented and leads to impaired postural and ambulation control and an increased risk of fall and injury (14). Gait and balance disorders can greatly affect daily life on a physical and psychological level and are therefore one of the leading causes of death among the senior citizens. Although medical conditions like arthritis and orthostatic hypotension are the common causes, most of the gait and balance disorders are multifactorial in origin and the consideration of both the contributing factors and the targeted interventions require a comprehensive evaluation (15). Deficits in executive functioning skills, which include a group of higher cognitive processes concerning one's ability to organize thoughts, prioritize tasks and make decisions, are associated with impairments in postural control, reduced gait speed, and increased falls risk (16, 17). Cognitive impairment and affective disorders or psychiatric conditions such as depression, anxiety, fear of falling, and sleep disorders have been also connected with balance dysfunctions and falls (18).

Due to the multifactorial nature of the majority of balance and gait disorders, there is a need to combine several interventions of treatment in order to restore and improve functional capacity (19). Gait disorders that are related to chronic medical conditions can be treated to some extent by medical and surgical interventions. Moreover, the use of home environment assessment and intervention, especially when integrated in a multifactorial program, can significantly reduce falls and improve the quality of living of the elderly (20). On the other hand, balance training, including exercise and physical therapy, is gaining ground in the treatment of balance and gait disorders. The joint American and British Geriatric Society guidelines (21) recommend that a suitable balance exercise programme is a crucial component for balance rehabilitation. Through customized balance physiotherapy intervention, persons with postural deficits who have experienced falls or are at risk of falling, perform individualized exercises daily in a safe environment. The protocols commonly used in these interventions include a first visit to the physiotherapist for an initial assessment and the creation of a treatment plan. The patient then visits the specialist on a weekly basis, while also performing some exercises and a walking program at home. During treatment, the therapist assesses the patient's condition and progress and thus makes the necessary modifications in the program (22). The exercises included in the treatment plan are usually performed at physiotherapy and rehabilitation centers. Nonetheless, recent findings show that interventions that include exercise sessions in the home environment of the patient seem to be quite effective and present higher adherence rates than group-based community sessions (23). Cognitive training has also directly demonstrated benefits on balance and gait parameters in older adult fallers, while treatment strategies for anxiety and depression incorporated in balance training programs have proven to be effective (24, 25).

However, to date, there is a lack of personalized solutions that have been proposed to improve physical activity and the engagement of people with balance disorders to an exercise and physiotherapy program. The challenges in providing effective balance physiotherapy coaching include lack of compliance, difficulty in proper exercise performance and limited access to specialized physiotherapists and balance clinics. Despite evidence, suggesting that combined cognitive and functional training results in better outcome compared to either in isolation, there has been limited carryover into clinical practice.

The overall objective of HOLOBALANCE is to develop and validate a new personalized hologram coach platform for virtual coaching, motivation and empowerment of the aging population with balance disorders. The central idea of the intervention is the training of the users through a customized and personalized program, which will be formed through the use of ambient and wearable sensors and augmented reality interaction methods. The project engages experts related to the treatment of balance disorders, including physiotherapists, Ear Nose Throat experts, neurologists, psychologists, and gerontologists, who have the ability to track users' progress through an expert panel, formulate a comprehensive treatment plan and customize it with the help of progressing learning algorithms.

The HOLOBALANCE platform consists of the following coaching components:

- A hologram based surrogate balance physiotherapist
  The benefits of the traditional methods of physiotherapy and exercise training are fading because of lack of compliance or missed sessions as a result of the limited access to specialized clinics. Moreover, training programs that are performed at home environment often present poor adherence and fail to motivate the users. In order to overcome these challenges, we propose a virtual coach with daily presence in user's home. The physiotherapist, in the form of a hologram, can monitor, and assess the activities and the performance during balance training, while motivating and empowering the user. The reasoning of the virtual coach is implemented by a set of wearable and ambient sensors. This allows for an objective assessment of exercise performance and real-time feedback. For the physiotherapist and other healthcare professionals, the use of this sensor technology allows for ongoing daily activity coaching regarding quality of exercise performance, physical activity levels, activities of daily living performance and compliance with goals in order to provide feedback, update of instructions and exercise progression on a real time basis, which may further motivate and enhance self-management of users.
- The augmented reality cognitive games and exergames combined with auditory exercises
  The inclusion of the cognitive games and exergames module aims to empower and motivate people during their balance physiotherapy through augmented reality gamification. It includes games that incorporate key design factors from the entertainment video game world and aim to place both physical and cognitive training in a pleasant, motivating and engaging context. On the other hand, the main goal for the auditory exercises is to provide training tasks that improve the patient's auditory memory and perception of speech in noise. The auditory training is conducted through a smartphone application.
- The physical activity planning component
  The sensor technology used during physiotherapy sessions allows for continuous monitoring of patients' levels of physical activity, their progress and their degree of compliance with the treatment plan. The healthcare professionals engaged in the project can have access to these data through a specially designed dashboard interface. They can use this interface to register new patients in the HOLOBALANCE system, store useful information and define or adjust the physical activity and physiotherapy plans (26). The Activity Planning Application (APA) module, which is the main user interface, plays a crucial part in the physical activity planning. It is a smartphone application that allows users to constantly monitor their training plan as well as their progress, both in terms of physical activity and interaction with all parts of the system. It fulfills the key motivational strategies of the platform in order to engage the users and maintain their interest in the use of the system. Toward this direction, the Virtual Communities (VCs) dashboard integrates with the application. The main purpose of the VCs dashboard is to manage the interaction of the patients with each other in a virtual community, where they can get in touch with other users of the HOLOBALANCE system, while being in touch with their clinicians. The authorized users of the dashboard, consisting of clinicians and local agent communities, can inform the patients about a variety of interesting issues and notify them about upcoming events. They can also satisfy a user's request to display a message or a suggested event in the community. The users' requests, the declaration of their willingness to attend an event and the display of the approved message and event list are performed through the APA that interacts with the VCs component.

**Figure 1:**
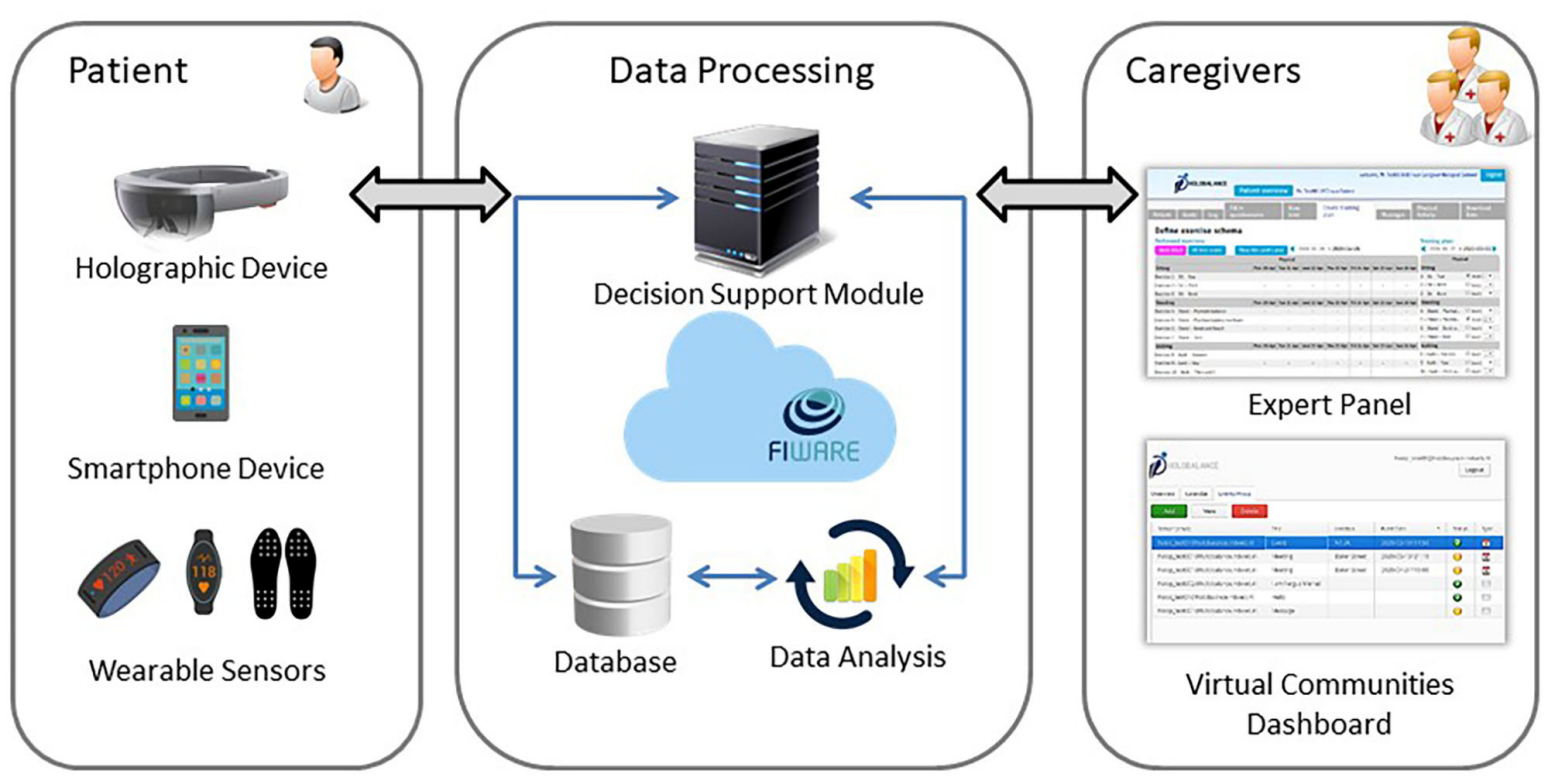
Outline of HOLOBALANCE architecture.

An outline of the HOLOBALANCE architecture is shown in Figure 1. The part of the system that is placed in-home environment near the user consists of IoT devices that enable the monitoring of user's actions, behavior changes and level of activity. A set of hardware-software interfaces provide a standardized and interoperable layer to communicate with different sensor devices and acquire their data. A FIWARE-Orion enabled communication module handles the interaction between the components that are placed in the user's home and the cloud infrastructure. The latter integrates all the required services for the interaction between its different modules and performs all actions related to data exchange, processing, advanced analytics estimation and representation. It is consisted of a REST/JSON API over HTTPS that provides generic database queries for Create, Read, Update, and Delete (CRUD) operations. Authorization, authentication and user management are also provided. The data repository of the cloud supports various database engines and types of data as well as data replication and push notifications through Firebase. The advanced data analytics module of the cloud infrastructure allow the deployment of machine learning and deep learning algorithms to execute condition evaluation and user behavior tasks on data collected from the devices. The results of this multilevel analysis are used to feed back the system and the interfaces available to the involved clinical experts, through which they have overall oversight of the intervention. The main goal of the proposed interoperable platform is to remove restrictions regarding the system development and the design of any potential application to specific hardware and software components. It promotes a system that can be easily deployed in different areas, shifting the effort from design time investments to the process of developing innovative telehealth applications with advanced analytics and more efficient treatment plans (27).

## The Holobalance Activity Planning Application

### Purpose

The APA is the main interface through which users communicate with the HOLOBALANCE platform. The main purpose of the application is for them to be able to constantly monitor their activity, as well as to determine to what extent they benefit from the use of the system. As poor adherence is one of the main challenges in long-term interventions, the application is intended to implement a significant part in inciting user engagement and motivation. Thus, all the design and implementation steps described in detail below were based on the objectives and the desired impact of the intervention, taking into account the characteristics and requirements of the target user group.

### User-Experience Design

Determining the necessary functions and data that an application should contain is the first important step in creating a pleasant and meaningful user experience. This process is guided by the clarification of the target group and the main objectives of the application. The APA is aimed at adults over the age of 65 who suffer from balance disorders. Its main purpose is to offer patients with the capability to monitor their activity and have an overview of their progress resulting from their interaction with the components of the HOLOBALANCE platform. For that purpose, a number of functionalities regarding the display of information and the provision of education have been added. Through the smartphone application, users are enabled to keep track of the key elements regarding their activity recorded by an activity tracker, at any time of the day as well as of how close they are to achieving the goal set in their treatment plan. Social interaction functionalities were also determined as a powerful method for enhancing user health behaviors. The users of APA can communicate with other members of the HOLOBALANCE virtual community through the social interaction component of the application. They are informed about upcoming events and interesting issues and can exchange their ideas and concerns.

User-experience design has a major impact in the motivation of the users and in the effectiveness of the application. Thus, it is crucial to design and implement an overall motivation strategy that enhances user engagement. The purpose of APA is to motivate users both in terms of their daily physical activity and their engagement with the various components of the HOLOBALANCE tele-rehabilitation system.

The HOLOBALANCE is a complex intervention with several interacting components. It aims to bring about a series of behavioral changes concerning the health, the balance and the well-being of users. Theories of behavioral change aim to provide a theoretical framework for the factors influencing behavioral change and motivation (9) and which in turn are related to the success or failure of an intervention. A large number of theoretical frameworks have been proposed to conceptualize health-related behavioral change (10). These frameworks vary in their degree of empirical support, and many have been developed for specific health domains which many not be relevant to the specific characteristics of the HOLOBALANCE intervention (11).

Thus, a systematic approach was adopted for the selection of the theoretical framework to be used in HOLOBALANCE, based on the following criteria:

- The framework should be comprehensive, in the sense that its components include not only the intentional and motivational elements resulting in intervention adherence, but also the interplay of these psychological factors with the physical means, or physical capability to execute the intervention. This is important because HOLOBALANCE is an intervention delivered through technology.
- The framework should have been empirically validated through meta-analysis reporting that interventions informed by the model lead to changed behaviors.
- The framework should have been previously applied to other interventions similar to HOLOBALANCE, as assessed by publications on the following: promoting adherence to interventions with computer-based technological components, intervention involving physical activity, and population target of older adults.

Based on the above, the Capability, Opportunity, and Motivation (COM-B) model of behavior was chosen (12). The model posits that the interaction between Capability, Opportunity and Motivation (COM) causes the changes in Behavior (B), where: Capability is the “individual's psychological and physical capacity to engage in the activity concerned,” Opportunity includes the “factors that lie outside the individual that make the behavior possible or prompt it” and Motivation includes Reflective Motivation (evaluations, intentions, and plans) and Automatic Motivation (emotions and impulses arising from learning and innate dispositions). The COM-B model incorporates the interplay between psychological and physical elements of the behavior through the components of physical capability (the physical ability of the person to engage in the target behavior), and physical opportunity (the environmental resources, including technology, that facilitate the target behavior).

This model has been developed as part of the Behavior Change Wheel (BCW), which is designed to help intervention designers move from a behavioral analysis of the problem to intervention design using the evidence-base. The BSW defines nine intervention functions (education, persuasion, incentivization, coercion, training, restriction, environmental restructuring, modeling, enablement) and seven policy categories (communication/marketing, guidelines. fiscal measures, regulation, legislation. environmental/social planning, service provision) and allows developers to identify systematically which of them could bring about behavioral change (28). Once the intervention functions and the policy categories are chosen, behavior change techniques and effective modes of delivery are identified.

The COM-B and BCW have already been used in areas relevant to the HOLOBALANCE intervention, including gamification of mobile health interventions (29, 30) and promoting adherence to hearing aids in older adults (31) while it has also been used in a recent meta-analysis to classify motivational interventions to promote exercise adherence for older fallers (32). After a detailed literature review of the parameters that promote physical exercise and exercise adherence, especially in the elderly, as well as after the feedback that was given in older adult workshops that were organized as part of the project, the motivation strategies of HOLOBALANCE were determined. These key strategies were related to the relevant components of COM-B and BCW models (33).

The motivational part of the APA is based on the overall motivational strategy of the project. Once the target behaviors and the appropriate intervention functions that will lead to behavioral changes were identified, according to the COM-B model, the specific Behavioral Change Techniques (BCTs) that the application will implement were defined. By BCT, we mean an observable, replicable, and irreducible component of an intervention designed to alter or redirect causal processes that regulate behavior; that is, a technique is proposed to be an “active ingredient” (e.g., feedback, self-monitoring, and reinforcement). BCTs can be used alone or in combination and in a variety of formats (34). The selection of techniques was made using the BCT Taxonomy (BCTTv1), which lists 93 BCTs with descriptions and examples of their application (35).

The motivation features that were derived from the above procedure and are included in the application can be categorized logically in informative, gamified, and social features (36).

#### Informative Features

These features allow the user to monitor their physical condition and progress in an easily accessible and understandable way. They are based on the fact that providing information to the user can itself be a powerful motivator for more effort. In the context of our application, the users can be informed at any moment about their daily step target, which is determined by the attending clinician, and about their longer-term general goals that have been formed in consultation with him/her. Their progress is displayed on the main screen when entering the application both in percentage and in numbers, while a card contains other useful information about their activity, such as the calories they burned and the distance they traveled. An arch chart has been chosen to visualize what part of the daily step goal has been fulfilled. It is also possible for them to monitor the long-term course of their activity through a 5-day chart. In this bar chart, the steps performed on each one of the past 5 days are represented by a bar, the color of which depends on whether the user has achieved the daily step target. Notifications are sent daily, informing and encouraging the users to complete their daily training plan and fulfill their goals. A message is displayed in the notification drawer and when tapped the user is redirected to a list with the exercises of the defined training program annotated based on whether they have already been performed or not. However, users are not only motivated when they are aware of their activity and progress but also when they receive general information and training about the goals and benefits of the intervention in which they participate. Through the functionalities that result from the integration of VCs dashboard with the application, the clinicians can provide easily accessible supportive educational material to users.

#### Gamified Features

Gaming methods are widely used in motivation strategies, as they enhance the maintenance of the user's interest and engagement in a pleasant way. This is the integration of methods encountered in computer games, since they are first adapted to the requirements and needs of the users of each application. Consequently, it is crucial for an intervention for senior citizens to account for the most important age-related issues. Elderly users are not familiar with gaming systems and gamified features that are included in non-gaming applications. The lack of experience they are likely to have with gaming can be a barrier to understanding the metaphors derived from digital games and therefore can hinder their engagement (37). In our implementation, easily accessible and simple gamified features that do not assume user's prior gaming experience were chosen. A number of badges are calculated and given to the user as rewards for achieving several goals. The calculation is performed individually, taking into account the treatment plan and the goals set separately for each user by the clinician. The collection of badges covers the activities of all the interventions that compose the HOLOBALANCE project and is based on the logic of positive feedback, rewarding the effort more than the performance. It includes a number of predefined badges as well as some badges that are initially locked and can be unlocked after several achievements. By introducing these unlockable features, the users are encouraged to remain engaged over a longer period of time.

As part of the healthy competition between the members of the project community, a leaderboard was integrated in the application. Through this component, which is reinitialized and updated every week, the users are able to see their weekly ranking between the other members of the community, without being able to see the names of the other competitors. The rankings are based on the users' badges. Specifically, possession of each of the badges is translated through an algorithm into the possession of “stars,” which is our unit of scoring.

#### Social Features

Social influence is a great source of motivation. Through social support and pressure the users are motivated to be better and maintain their interest in the use of the system. Communication and interaction between users of the system is mainly through the functions of the Cs dashboard, which are accessible through the application. Specifically, users can exchange messages, be informed about events planned in their area as well as suggest the organization of an event. In this way, they are given the opportunity to communicate their concerns, build relationships and feel part of a team with a common goal, which is to encourage and motivate them. The leaderboard component, which was described above, can also be mentioned among social features, as it allows users to share their performance with others and compare their results.

The motivational features of the application and the BCTs to which each one corresponds are summarized in Table 1.

**Table 1:** The motivational features of the application and their correspondence with the Behavioral Change Techniques Taxonomy (BCTTv1).

**Motivational features of APA**	**COM-B component**	**BCT Label (BCTTv1)**	**BCT definition**
Daily target/general goal/progress chart	Psychological capacity, reflective motivation	2.2 Feedback on behavior	Monitor and provide informative or evaluative feedback on performance of the behavior (e.g., form, frequency, duration, intensity)
		2.7 Feedback on outcomes of behavior	Monitor and provide feedback on the outcome of performance of the behavior
Notifications	Physical opportunity	7.1 Prompts/cues	Introduce or define environmental or social stimulus with the purpose of prompting or cueing the behavior. The prompt or cue would normally occur at the time or place of performance
Educational material through virtual communities	Psychological capacity	4.1 Instruction on how to perform a behavior	Advise or agree on how to perform the behavior (includes “skills training”)
		5.1 Information about health consequences	Provide information (e.g., written, verbal, visual) about health consequences of performing the behavior
		5.3 Information about social and environmental consequences	Provide information (e.g., written, verbal, visual) about social, and environmental consequences of performing the behavior
		5.6 Information about emotional consequences	Provide information (e.g., written, verbal, visual) about emotional consequences of performing the behavior
Badges/achievements	Automatic motivation	10.4 Social reward	Arrange verbal or non-verbal reward if and only if there has been effort and/or progress in performing the behavior (includes “positive reinforcement”)
		10.5 Social incentive	Inform that a verbal or non-verbal reward will be delivered if and only if there has been effort and/or progress in performing the behavior (includes “positive reinforcement”)
Leaderboard	Social opportunity	6.2 Social comparison	Draw attention to others' performance to allow comparison with the person's own performance
Messages/events through virtual communities	Reflective motivation, psychological capacity	3.1 Social support (unspecified)	Advise on, arrange or provide social support (e.g., from friends, relatives, colleagues, “buddies” or staff) or non-contingent praise or reward for performance of the behavior It includes encouragement and counseling, but only when it is directed at the behavior

### User-Interface Design

Designing a usable, efficient and enjoyable mobile application requires the combination of emotional and cognitive components. The basic usability and aesthetics principles should be satisfied and user needs and preferences should be constantly in the forefront throughout the process. As for the group of elderly users, the perception of their negative attitude toward technology often results in a lack of emphasis on their special needs during the design procedure. There is a general mismatch between perception about elders' needs and their actual needs, and a misinterpretation of elders' needs and demands by the non-elder users (38). The fact that people today live more and want to be active and independent as well as the fact that they are becoming more and more familiar with the technology and the benefits it offers them in their daily lives, makes it imperative to design technologies that will satisfy their needs.

The APA was created in order to be used by elderly people, therefore in the part of its design emphasis was placed on the needs and requirements of this age group. When aging, there are three main problem categories: sensory problems, motor problems, and cognitive problems (38). Below is an extensive description of each of the above categories of challenges, as well as the design decisions made in the application to address them:

- **Sensory problems**
  One of the characteristics of old age is the weakening of all the senses, but mainly those of hearing, sight and touch. The majority of people over the age of 40, regardless of gender, begin to show some degree of progressive hearing loss as time goes on. This decline is greater at higher frequencies (39). APA does not include any audio components or ring tones in its context. However, daily notifications are sent through the application. In order to ensure as much as possible that the user will be notified, even if there is difficulty in hearing the alert sound, vibration and light signals have been added when designing the notification module. Regarding the sensor of sight, older people present impairments regarding the breadth of visual field, the visual processing speed and the perpetual flexibility (38). They have difficulties in reading text in menu and text messages especially when font is not gothic, and when color contrast between text and background is not obvious (40). The application presented in this paper uses big font, big buttons and obvious color contrast. It includes many simple icons that represent different concepts, but there is a concern for their simultaneous description in the form of text. Thus, any difficulties related to reading the text or understanding the icons are addressed.
- **Motor problems**
  Movement control and manual dexterity is affected by aging, especially when diseases like multiple sclerosis or Parkinson's disorder make their appearance. Older people need more time to respond to a movement task and their moves are less precise, mainly due to a decrease in the muscle mass and strengths (38). They have difficulty in differentiating short push and long push on one button and they type slowly (40). APA is designed to include the absolutely necessary number of buttons, each of which performs a single function. Spacing is as large as possible, always following the design properties of each component, so as to minimize the possibility of pressing in the wrong place. Textual feedback is given when the effect of a movement is not clear to the user.
- **Cognitive problems**
  The deficits concerning the cognition level of seniors need to be carefully considered when designing a mobile application. Attention, memory and decision-making ability are affected by aging, thus affecting human-machine interaction. Elders find it difficult to learn and understand new procedures or large amount of information at once, perform difficult tasks and solve problems (41). Regarding the above, the navigation process of APA has been kept simple and austere. Only task-relevant information is displayed, avoiding parallel material and tasks. Pop-up windows and other dialog view components are displayed longer than usual in smartphone applications, in order to facilitate their processing by the user. Memory-requiring actions and tasks that assume either user's familiarity with mobile applications or knowledge of metaphors and interaction techniques have also been avoided. Notifications are sent through the application and serve as reminders and memory aids for the users, informing them about their training program.

### Technical Design

The application presented in this document was developed for Android platform. Most functionalities are implemented through Android software development kit and the use of external libraries has been minimized. Model-View-View-Model pattern was chosen as the main software architectural pattern in order to keep a reusable and testable code.

The APA is part of a complex and multifactorial intervention and it is necessary to retrieve and process data recorded from other technological modules of the system. The information about their daily activity recorded by the Fitbit activity tracker, as well as performance data regarding other platform's components are retrieved from the HOLOBALANCE backend infrastructure. The application also retrieves data about the posts and the events that should be displayed in the newsfeed page, where users can communicate and get informed about interesting topics. These data are available through the VCs interface, where the authorized clinicians and local agent communities can define the content of the newsfeed page that will be displayed to the profiles of a group of patients.

**Figure 2:**
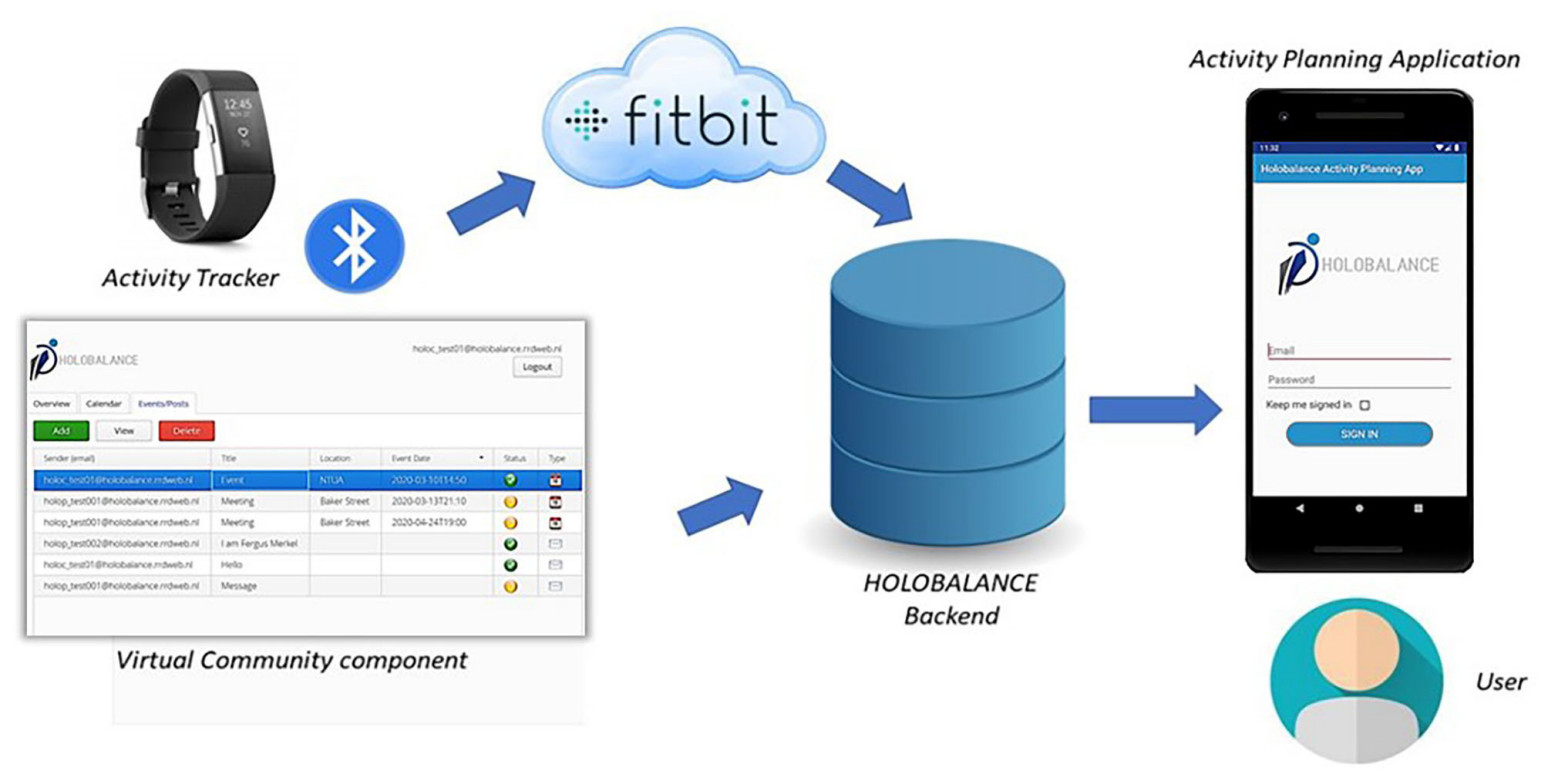
Activity Planning Application in the HOLOBALANCE system.

The communication between APA and the other modules uses HTTP protocol via JSON formatted messages in RESTful manner. As a result, the application receives real-time feedback concerning the activity and the training plan of the patient, as well as the content of the social component. Regarding the rewarding system of the application, data recorded by the activity tracker and results from physiotherapy, cognitive and auditory training are needed. APA consumes these data from the HOLOBALANCE cloud infrastructure, where they are stored. The interactions between the APA and the other modules of the system are shown in Figure 2.

### Evaluation

The design and development of the APA followed a human-centered design approach. That is, through an iterative development process, the APA requirements were specified, new mock-ups and prototypes were implemented, and were then evaluated by the end users. To analyze the requirements and evaluate the mock-ups, several semi-structured focus groups and interviews were performed across three European countries (UK, Germany, and Greece). After each iteration, new insights have evolved which led to re-prototyping of the concepts and content elements.

Following this approach could ensure the user experience requirements of the final product and an easy-to-use tool that communicates harmoniously with the rest of the system and serves the purposes and strategies of the overall intervention. Details about the participants of the focus-groups and interviews that were performed as well as the evaluation protocol that was followed are presented in Table 2.

**Table 2:** Information about the participants and the evaluation protocol of the semi-structured focus groups performed in UK, Germany, and Greece.

	**Focus group location**	**Protocol**	**Participants**
Iteration I	London (UK)	1. Welcome, introduction, and obtaining participants' consent for participation 2. Demographic questionnaires 3. Demonstration of the 1st mockup of APA 4. Q&A with participants and clinical, technical, and usability experts 5. APA usability questionnaire (modified SUS) and open-ended questions	1. *N* = 47 2. 29 females (63%) 17 males (37%) 3. Age: 60–84 years (*M* = 73.61, *SD* = 6.12, Mdn = 72.5) 4. *N* = 13 (28%) had balance disorder 5. 94% use computers daily 6. Confidence in using technologies = 3.32 (*SD* = 0.91, Mdn = 3) 7. 60% familiar with social media
Iteration II	Freiburg (Germany) and Athens (Greece)	1. Welcome, introduction, and obtaining participants' consent for participation 2. Participants used all Holobalance modules including APA for ~15 min 3. Q&A with participants and clinical, technical, and usability experts 4. Questionnaires (Demographic, usability, etc.) and open-ended questions	1. *N* = 24 2. Freiburg: *N*= 8, Athens: *N* = 16 3. 21 females (63%) 3 males (12.5%) 4. Age: 60–84 years (*M* = 71.42, *SD* = 7.99, Mdn = 72) 5. *N* = 12 (50%) had balance disorder 6. 58.33% use computers daily 7. Confidence in using technologies = 2.33 (*SD* = 1.34, Mdn = 2.5)

The initial requirements were defined through interviews and focus groups with the project's clinical experts. Based on these requirements, the first mock-up of APA was developed. This mock-up was then evaluated through a semi-structured focus group with older adults in London. During the focus group, participants could ask their questions, discuss the presented mock-up with the technical and clinical experts, and finally express their opinions through questionnaires and direct conversations with clinical, technical, and usability experts.

Forty-seven older adults (29 females) aged between 60 and 84 years (*M* = 73.61, *SD* = 6.12, Mdn = 72.5) participated in the focus group, 13 (28%) of whom had balance disorder. Regarding their familiarity with technology, most of them (94%) claimed that they use computer systems (including smart phones, tablet PCs, etc.) daily. On a scale of 1 (very bad) to 5 (very good), their confidence in using technologies was rated on average 3.32 (*SD* = 0.91, Mdn = 3). They were also asked about their experience in using social media. The average (60%) claimed that they have used social media and Facebook was named as the most frequently used application.

After the demographic questionnaire, the APA was presented and explained to the participants. In order to estimate the usability aspects of the APA, a modified version of the System Usability Scale (SUS) (42) was given to the participants. It consisted of the five most important items of the SUS for our case. Its four items were rated on a scale of 1 to 5 representing a range of Strongly Disagree to Strongly Agree with the statement. The Assistance item, consisted of multiple choice questions and was served to estimate how many percent of the participants needed what type of assistance. The items of this questionnaire and the participants' responses are presented in Table 3.

**Table 3:** Means (standard deviations), medians, and percentage of the responses to a modified version of the SUS questionnaire.

**Item**	**M (*SD*)**	**Mdn**
I would like to use this system frequently if it meant that it would reduce my risk of falling	3.75 (1.28)	4
I think this system would be easy to use	3.46 (1.32)	3
I would imagine that most people would learn to use this system very quickly	3.53 (0.98)	3
I would feel very confident using this system	3.73 (1.12)	4
I think that I would need assistance to be able to use this system. If yes, what type?	**Percentage (%)**	
No assistance	30.6	
Physical assistance	5.6	
Technical assistance	50	
Educational assistance	16.7	
Medical assistance	2.8	

**Figure 3:**
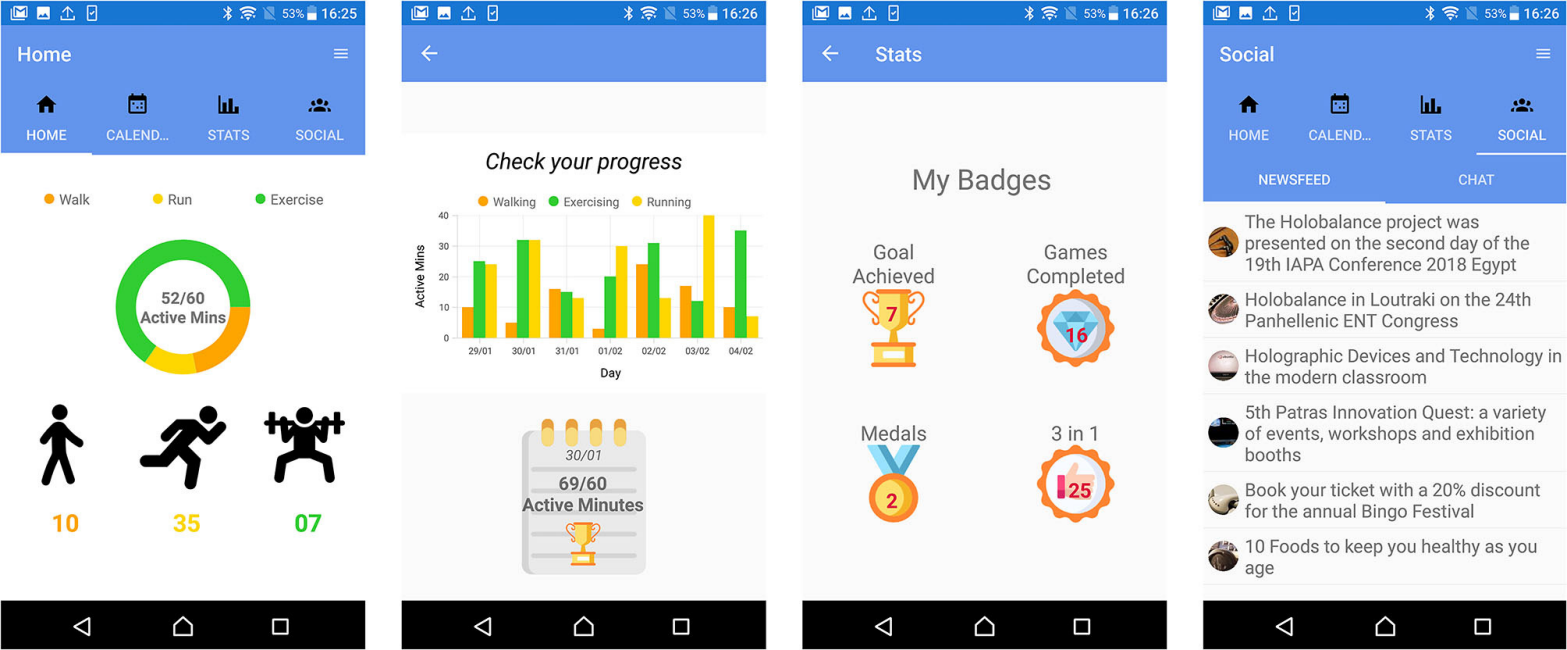
Mock-ups of the Activity Planning Application.

Focus groups and interviews were also performed in Freiburg (Germany) and Athens (Greece). We interviewed in total 24 older adults (21 female) between 60 and 84 years old (*M* = 71.42, Mdn = 72, *SD* = 7.99), from whom eight were in Freiburg and 16 in Athens. Half of them believed that they have balance disorder. About two-thirds (66.66%) had access to broadband internet at home and used a computer (including smart phones, tablet PCs, etc.) on a daily (58.33%) or weekly (4.16%) basis. However, the other third (37.5%) reported that they never use any computer system. Moreover, the participants' average confidence with technology on a scale of 1 (very bad) to 5 (very good) was 2.33 (*SD* = 1.34, Mdn = 2.5). Among the other components of the HOLOBALANCE platform, mock-ups of the APA were presented to the participating older adults (Figure 3). They were then asked to comment on their experience of interacting with the application and to share their ideas, comments, and impressions. The followings are some examples of their comments:

- “You need to prepare a manual for the application”
- “It is simple, I like it”
- “I do not know if the competition between users would be a good idea”
- “I would like to see the others' progress”
- “I am not familiar with smartphones”
- “I don't have a mobile phone and don't know how to handle it”
- “It is good to know for how long I was active during the day”
- “I can see the letters but maybe the buttons could be bigger”
- “I do not know if I want to chat with the other members.”

The above results from the focus groups organized in London, Freiburg, and Athens showed that the participants were neutral about using the APA. A large number of participants (about 50%) noted that they require technical assistance to use it. Most found the application simple and could navigate without problems. Some, however, suggested changes in the design that would make it easier to use, such as using bigger buttons and a larger font size. More detailed evaluation outcomes, especially regarding other aspects and modules of the Holobalance system can be found in (43, 44).

**Figure 4:**
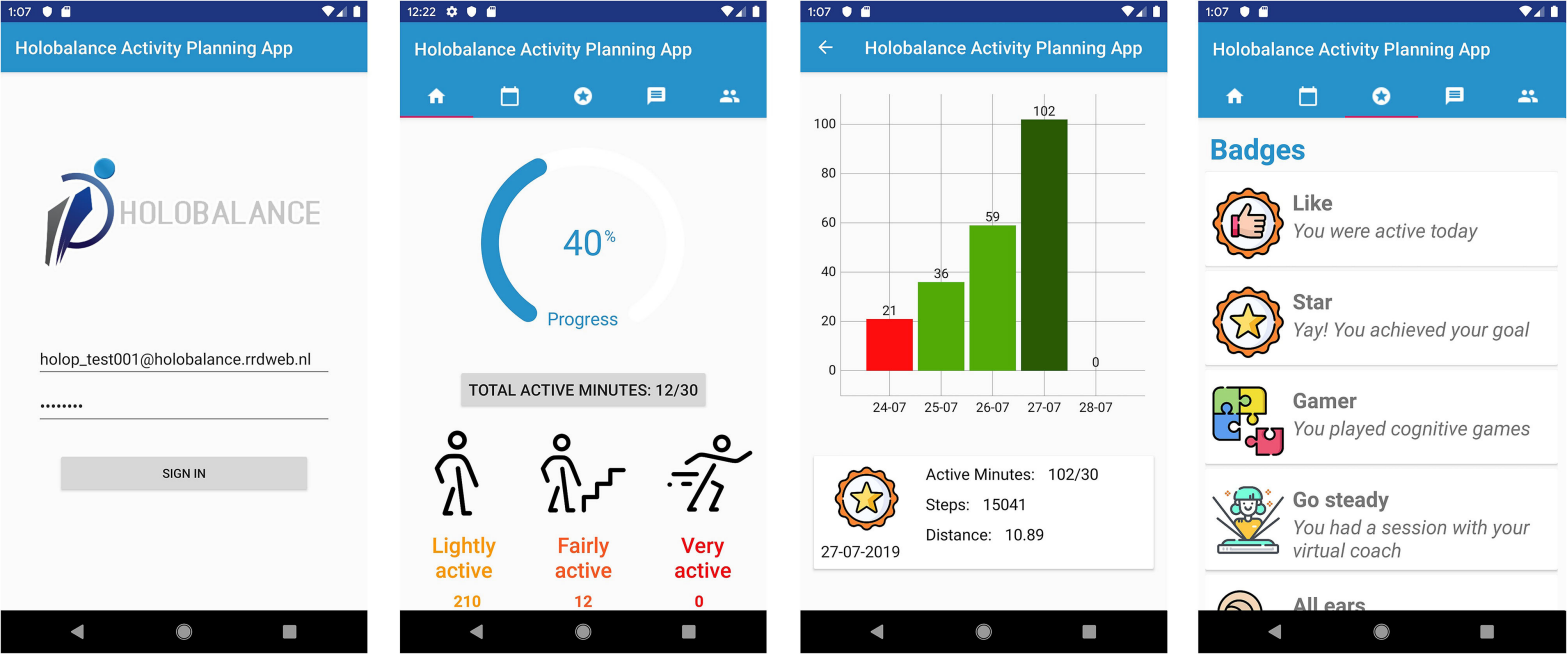
First version of the Activity Planning Application.

**Figure 5:**
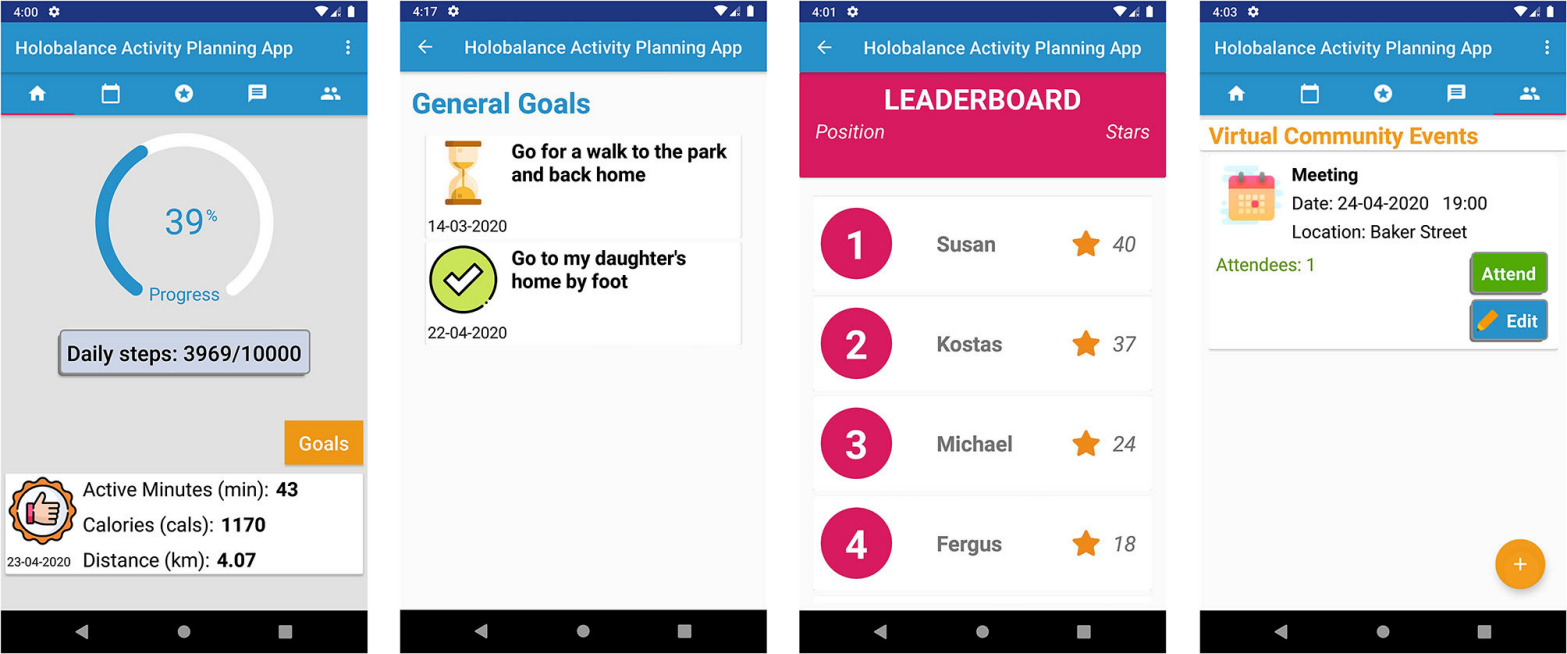
Final version of the Activity Planning Application.

Based on the feedback from end users as well as from the clinical experts of the HOLOBALANCE project, different versions of the APA were created, as many features were evaluated and updated (Figures 4, 5). The evaluation of the current version's adoption and acceptance will be conducted to the target users involved in the proof of concept at the pilot studies of the project. Four pilot sites were determined and each of them will recruit 20 participants and 20 control subjects. In total 80 participants in the intervention group and 80 in the control group will be recruited. Qualitative and quantitative measures will be used to process the results of these pilot studies and validate the use of the APA.

## Discussion

The rapid growth of mobile technology in recent years has paved the way for the development of innovative healthcare interactive systems. mHealth applications are widely used either as stand-alone or as part of a more comprehensive system for the purpose of disease management and healthy life-style. Smartphones are often involved in interventions for the elderly, as they are more familiar and prefer them over other technologies.

In this article, a smartphone application integrated in a complex intervention to support older people with balance disorders is described. The process of the application's design and development was human-centered and special emphasis was placed on the requirements of the target user group. The first mock-up interfaces were defined through interviews and focus groups with the project's clinical experts. They were subsequently evaluated in semi-structured focus groups and interviews that were performed across three European countries. The results were encouraging, as participants found the application simple to use and easy to navigate. However, they also pointed out their lack of familiarity with technology and their need for technical support. The feedback on the design features and the functionalities of the application led to the revision of several content elements, following an iterative development approach. The current version will be evaluated at four pilot studies, which will recruit a total of 160 people, divided equally into one intervention group and one control group.

## Data Availability Statement

The original contributions presented in the study are included in the article/Supplementary Material, further inquiries can be directed to the corresponding author/s.

## Ethics Statement

This study involved human participants and was reviewed and approved by the Ethics and Deontology committee of Hippokration General Hospital, Athens (reference number: 9769/24-6-2019), by the Ethics Committee of the Medical Association of Westphalia-Lippe and Westphalian Wilhelms University (reference number: 265/19), by Health Research Authority and Health and Care Research Wales (IRAS project ID: 248101) and by the Biomedical & Health Sciences, Dentistry, Medicine and Natural & Mathematical Sciences Research Ethics Subcommittee of King's College London. The Trial Registration Number of the study is NCT04053829 (https://clinicaltrials.gov/ct2/show/NCT04053829). The patients/participants provided their written informed consent to participate in this study.

## Author Contributions

TA, AA, and IK made substantial contribution to the design and the development process of the smartphone application. D-EB, GG, and SC made substantial contribution to the design of the motivation model. FM made substantial contribution to the evaluation of the application. SP and DK had general supervision of the development stage of the application. All authors contributed to revising the manuscript.

## Conflict of Interest

The authors declare that the research was conducted in the absence of any commercial or financial relationships that could be construed as a potential conflict of interest.

## Footnotes

^1^ https://holobalance.eu/
